# Ganoderma lucidum inhibits proliferation of human ovarian cancer cells by suppressing VEGF expression and up-regulating the expression of connexin 43

**DOI:** 10.1186/1472-6882-14-434

**Published:** 2014-11-05

**Authors:** Shuyan Dai, Jingjing Liu, Xiaofei Sun, Ning Wang

**Affiliations:** Department of Obstetrics and Gynecology, The Affiliated Shengjing Hospital, China Medical University, 36 Sanhao Street, Heping district, Shenyang 110004 People’s Republic of China

**Keywords:** Ganoderma lucidum, Ovarian cancer, Real-time PCR, Immunohistochemistry, Vascular endothelial growth factor (VEGF), Connexin 43 (Cx43)

## Abstract

**Background:**

*Ganoderma lucidum* (*G. lucidum,* Reishimax) is an herbal mushroom known to have inhibitory effect on tumor cell growth. However, the molecular mechanisms responsible for its anti-proliferative effects on the ovarian cancer have not been fully elucidated.

**Methods:**

Human ovarian cancer cells HO 8910 (HOCC) and human primary ovarian cells (HPOC) were treated with *G. lucidum*. Effects of *G. lucidum* treatment on cell proliferation were studied by MTT assay. The expression of vascular endothelial growth factor (VEGF) and connexin 43 (Cx43) were measured by immunohistochemistry and real time polymerase chain reaction. To study the molecular mechanism of CX43 mediated anti-tumor activity, small interference RNA (siRNA) was used to knockdown Cx43 expression in HOCC.

**Results:**

*G. lucidum* treatment resulted in reduced proliferation of HOCC. Inhibition of proliferation was accompanied by a decrease in VEGF expression and increase in Cx43 expression in the cancer cells. The extent of immune-reactivity of Cx43 or VEGF in cancer cells were correlated with the concentrations of *G. lucidum* used for treatment*.* Furthermore, knockdown of Cx43 expression in HOCC abrogated the effect of *G. lucidum* on cell proliferation without alteration of *G. lucidum*-induced attenuation of VEGF expression.

**Conclusions:**

*G. lucidum* inhibits ovarian cancer by down-regulating the expression of VEGF and up-regulating the downstream Cx43 expression. *G. lucidum* may be a promising therapeutic agent for the treatment of ovarian cancer.

## Background

Ovarian cancer is the most frequent cause of cancer related death in women [1]. The high mortality can be attributed to late diagnosis and lack of effective treatment especially at later stages of the disease. Dried powder of a medicinal mushroom *Ganoderma lucidum* has been used in Chinese traditional medicine for over two thousand years. The mushroom is also used as a dietary supplement in other parts of the world. Extracts of the mushroom are known to have several biological properties including anti-tumor activities on different cancer cells lines.

The anti-cancer activity of *G. lucidum* is observed at different stages of carcinogenesis. Anti-cancer activity of the mushroom includes cell cycle arrest, induction of apoptosis and autophagy, and suppression of metastasis and angiogenesis [2]. *G. lucidum* has been shown to exert multiple anti-tumor effects on ovarian cancer cells and enhance the sensitivity of epithelial ovarian cancer (EOC) cells to cisplatin [3, 4]. However, the molecular mechanism responsible for the inhibitory effects of *G. lucidum* on the ovarian cancer has not been fully elucidated.

Vascular endothelial growth factor (VEGF) is an important regulator of vascular endothelial cell functions during tumor growth. Ovarian cancer cells secrete large amounts of VEGF, which plays a crucial role in the accumulation of ascites fluid, angiogenesis and tumor induced immunosuppression in ovarian cancer patients [5]. Increased expression of VEGF is also associated with poor prognosis in ovarian cancer patients [6, 7]. Gap junction is an important cell to cell communication structure playing an important role in physiological functions like cell differentiation, cell growth and tissue homeostasis. Several second messengers, small metabolites, and peptides for basic cell physiological activities are transported through the gap junctions [8]. Several carcinogens were shown to decrease the expression of connexins [9], while enhancement of connexin function had an inhibitory effect on the growth of cancers [10, 11]. The gap junction gene connexin 43 (Cx43) shows tumor-suppressing effects on various tumors [12]. Decrease in the expression of Cx43 was shown to decrease the sensitivity of ovarian cancer cells to chemotherapeutic agents. However the molecular mechanism of Cx43 on suppression of ovarian cancer is not known yet. Some studies have indicated that human ovarian surface epithelial cells and surgical specimens of normal ovary exhibit extensive Cx43 expression, whereas Cx43 expression in ovarian adenocarcinomas is nearly absent. These findings suggest that the loss of gap junctions and Cx43 are associated with a neoplastic process [13, 14]. Loss of gap junctional intercellular communication (GJIC) is critical for tumor progression because it allows the cells to escape growth control [15]. A defect in posttranslational phosphorylation of Cx43 protein is associated with low expression of the Cx43 gene which may be responsible for the GJIC deficiency in AB1 cells. Increased expression of Cx43 by gene amplification may restore this phosphorylated Cx43 protein and reestablish GJIC [16]. It has been reported that VEGF can stimulate the expression of Cx43 in cardiac myocytes [17, 18]. *G. lucidum* extracts are known to inhibit the expression of VEGF in a variety of cancer cell lines but its effect on Cx43 is not known. In the present study, we tested the effect of *G. lucidum* on amelioration of ovarian cancer and its effect on the expression of VEGF and Cx43.

## Methods

### Materials

#### Reagents

Cell culture reagents were purchased from Invitrogen (Carlsbad, CA). *G. lucidum* (Reishimax) was purchased from Pharmanex Guoyao Co. (Wuhan, China). This product was a standard extract of *G lucidum*, containing 6% triterpenes and 13.5% polysaccharides. The same product has been demonstrated to suppress growth of breast cancer cells [19], prevent breast-to-lung cancer metastasis [20], inhibit adipocyte differentiation, stimulate glucose uptake and activate AMPK [21]. Stock solution was prepared by dissolving the sample in sterile water at a concentration of 50 mg/ml and stored at 4°C, as described earlier [19].

#### Cell culture

Human ovarian cancer cells HO 8910 (HOCC) were purchased from Shanghai Bluegene Biotech CO., LTD (Shanghai, China). Human primary ovarian cells (HPOC) were purchased from the Institute of Biochemistry and Cell Biology (Shanghai, China). All cells were cultured in RPMI 1640 supplemented with 10% fetal bovine serum, penicillin (100 IU/ml), and streptomycin (100 μg/ml).

### HOCC and HPOC proliferation assay

HOCC and HPOC were harvested using 0.05% trypsin. Cells were suspended (40,000 cells/ml) in DMEM with 20% FBS, plated onto gelatinized 96-well culture plates (0.1 ml/well), and incubated at 37°C and 5% CO2 for 24 hours. The media was replaced with 0.1 ml of DMEM and 5% FBS with or without the addition of 10 ug/ml *G. lucidum* and incubated for 24, 48 and 72 h, at 37°C, 5% CO_2_. Cell proliferation was determined using MTT assay, mRNA expression and protein levels for Cx43 and VEGF were measured by real time PCR and Western blotting.

### Small interference RNA (siRNA) transfection

Cx43 siRNA and scrambled siRNA siRNA were designed and synthesized by Wuhan Genesil Biotechnology Co (Wuhan, China). The sequences for siRNAs were as follows: Cx43 siRNA: GCTGGTTACTGGCGACAGA; scrambled siRNA: TTCTCCGAACGTGTCACGT [22]. HOCC cells (40,000 cells/ml) in DMEM with 20% FBS, were plated on gelatinized 96-well culture plates (0.1 ml/well), and incubated at 37°C and 5% CO2 for 24 h. The media was replaced with 0.1 ml of DMEM and 5% FBS with the addition of 10 ug/ml *G. lucidum.* Cx43 siRNA (40 nmol/L) or scrambled siRNA was transfected into the cells using Lipofectamine 2000 reagent (Invitrogen, Carlsbad, CA, USA) according to the manufacturer’s instructions. The dose of Cx43 siRNA was selected based on a previous study which resulted in maximal knockdown of Cx43 expression in human aortic endothelial cells [22]. HOCC cells treated with 10 ug/ml *G. lucidum* alone served as control. After 72 h incubation at 37°C and 5% CO2, Cell proliferation was determined using MTT assay, mRNA expression and protein levels for Cx43 and VEGF were measured with real time PCR and Western blotting.

### Real time PCR

Real time PCR was performed using a Stratagene Mx3005P® QPCR System instrument (La Jolla, CA, USA) according to manufacturer’s protocol. RNA was isolated from HOCC and HPOC, followed by cDNA synthesis and data analysis as described previously [23]. Primers used for the real time qPCR were as following: Cx43, 5′-GGGTGACTGGAGCGCCTTAG-3′and 5′-TTATCTCAATCTGCTTCAAG-3′. VEGF, 4, 5′-AAGGAAGAGG AGAGGGGGCC-3′ and 5′- CTCCTCCTTCTGCCATGGGTG -3′; GAPDH, 5′-CCCTTCATTGACCTCAACTAC-3′ and 5′-CCACCTTCTTGATGTCATCAT-3′. GAPDH was used as an internal control.

### Western blotting

HOCC and HPOC cells were homogenized in RIPA buffer (150 mM Sodium chloride, 1% NP-40, 0.5% sodium deoxycholate, 0.1% SDS, 50 mM Tris-HCl (pH 8.0) containing protease inhibitor (Complete Mini Roche). After centrifugation the supernatants were boiled and mixed with an equal volume of sample buffer. Proteins were separated by SDS–PAGE and transferred to a polyvinylidene difluoride membrane (Millipore). The membranes were blocked with 5% skim milk in TBST (10 mM Tris (pH 7.5), 100 mM NaCl and 0.1% Tween 20) and incubated with primary antibodies 1 in TBST with 0.5% skim milk overnight at 4°C. The membrane was treated with primary antibodies and horseradish peroxidase-conjugated goat anti-mouse immunoglobulin G antibody (1:3000) (Amersham Biosciences) as secondary antibody. Immunoreactive bands were visualized by ECL (GE Healthcare) and quantified by densitometry with Image J software 1.45 (NIH, Bethesda, USA).

### Immunohistochemistry

Goat anti-mouse VEGF antibody (R&D Systems Inc. Minneapolis, MN. USA) and the goat anti-Cx43 antibody (Santa Cruz Biotechnology Inc., Santa Cruz, CA, USA) were used for immunohistochemical staining for VEGF AND Cx43. The HOCC and HPOC were fixed and processed in 10 mM of sodium citrate buffer (pH 7.6) in a microwave oven for four min twice at 70% power. Endogenous peroxidase was blocked by 5% hydrogen peroxide for 5 min. Nonspecific binding sites were blocked by 2% normal horse serum for 20 min. The samples were incubated with the primary antibodies for 60 min. Immunoreactivity was visualized by using DAKO Envision HRP System (1:10,000 dilution) (DAKO, Carpinteria, California, USA). Immunoreactivity was quantitatively evaluated using NIH image program. A score of 0–300 was calculated as the product of the intensity score and the percent of immunoreactivity.

### Statistical analysis

The association between the levels of immunoreactivity of Cx43 or VEGF and the concentration of *G. lucidum* was compared by one-way analysis of variance (ANOVA). The post hoc test used was Fisher’s protected least significant difference (LSD) test. Spearman's rank correlation coefficient was used to identify the strength of correlation between the levels of the Cx43 or VEGF and the concentration of *G. lucidum*. Data was analyzed using Statview 5.0 software (Abacus systems, Berkley CA) with a *p* value of <0.05 accepted as significant.

## Results

### HOCC and HPOC growth curve

**Figure 1:**
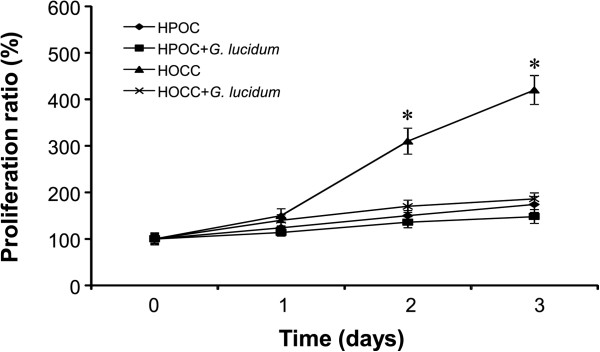
**Growth curve of HOCC and HPOC in DMEM with 5% BCS or with addition of 10 ug/ml** ***G. lucidum*** **.** Proliferation ratio (%) indicates the cell proliferation rate at other times compared with that at 0 h when cell numbers are considered as 100%. n = 5, Data are shown as mean ± S.D. *P < 0.05 vs HOCC + G. lucidum.

The HOCC and HPOC were cultured in DMEM with 1 5% FBS for 24, 48 and 72 h. The results for cell proliferation are shown in Figure 1. *G. lucidum* inhibited the growth of HPOC by less than 10% while it inhibited the growth of HOCC by up to 60% after 3 days of culture. These results suggest that *G. lucidum* effectively prevents the cell proliferation in HOCC.

### *G. lucidum*increases Cx43 mRNA expression and reduces VEGF mRNA expression

**Figure 2:**
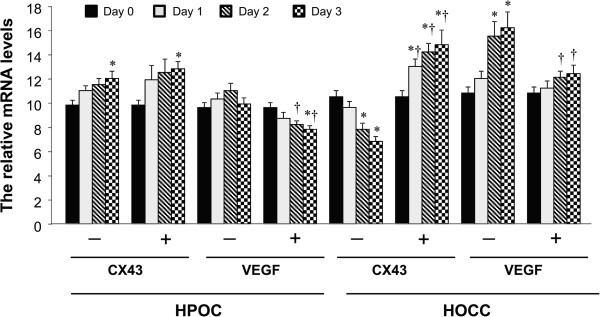
**The relative levels of Cx43 and VEGF mRNA in HOCC and HPOC.** "-" stands for untreated group (control). "+" stands for *G. lucidum*-treated group at dose of 10 ug/ml. Data are shown as mean ± S.D. of three independent experiments. All the cells were treated with *G. lucidum* for successive 3 days. *P < 0.05 vs respective day 0; †P < 0.05 vs corresponding untreated group.

The expression of Cx43 mRNA increased after 3 days of culture (P <0.05) in both untreated and *G. lucidum*-treated groups, compared with day 0 in HPOC cells (Figure 2). The VEGF mRNA expression in untreated group remained constant during 3 days of culture. However, VEGF mRNA expression in *G.lucidum*-treated group was found to be significantly reduced on day 3 in the HPOC cells. The expression of Cx43 mRNA decreased in the untreated group at day 2 and day 3 in HOCC cells. In contrast, the Cx43 mRNA expression in *G. lucidum*-treated group significantly increased from day 1 and remained at a high level on day 3. The VEGF mRNA expression in untreated group significantly increased by day 2 and continued to increase on day 3. However, no significant change in VEGF mRNA expression was observed in *G. lucidum*-treated group during the 3 days of culture. These data indicate that *G. lucidum* treatment effectively increases Cx43 mRNA expression and inhibits VEGF mRNA expression in HOCC but not in HPCC.

### *G. lucidum*increases Cx43 protein expression and decreases VEGF protein expression

**Figure 3:**
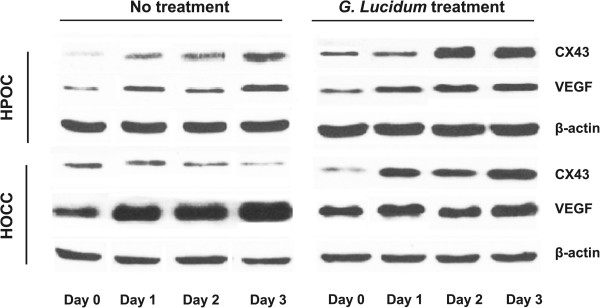
**Representative Western blots for Cx43 and VEGF in HOCC and HPOC treated with or without** ***G. lucidum*** **.**

**Figure 4:**
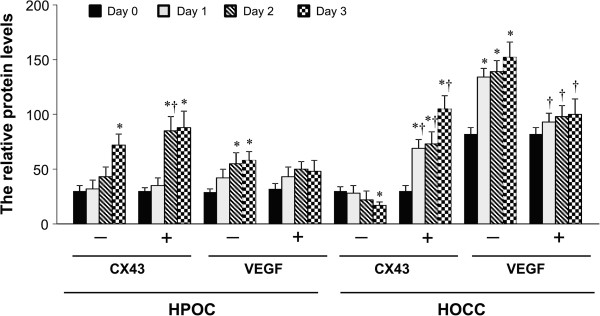
**Quantitative comparison of protein levels for Cx43 and VEGF in HOCC and HPOC treated with or without** ***G. lucidum*** **.** Data are shown as mean ± S.D. of three independent experiments. *P < 0.05 vs respective day 0; †P < 0.05 vs corresponding untreated group.

In untreated groups, the expression of Cx43 protein increased in HPOC, whereas it decreased in HOCC during 3 days of culture (Figures 3 and 4). The VEGF protein expression increased in both HPOC and HOCC at day 3. In treated group, the Cx43 protein expression was significantly augmented in both HPOC and HOCC at day 3. The increase in Cx43 protein expression was more pronounced in HOCC compared with HPOC. As expected, the VEGF protein expression was efficiently suppressed in both HOCC and HPOC treated with *G lucidum*. Thus, these results are consistent with mRNA data from our study and other report in the ovarian cancer cell [14], suggesting that HOCC are sensitive to *G. lucidum* treatment.

### The effects of *G. lucidum*on the protein levels of Cx43 and VEGF in a dose-dependent way in HOCC

**Figure 5:**
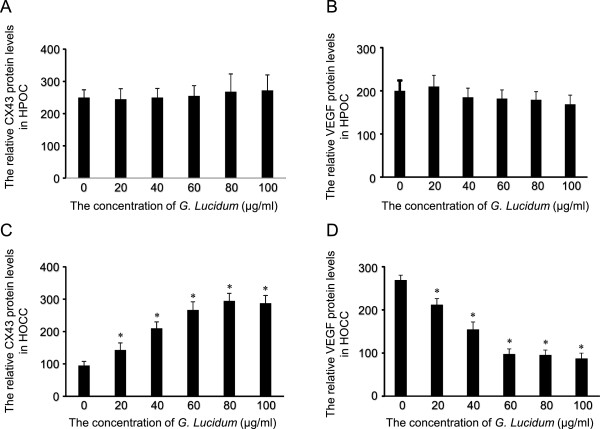
**Dose-dependent effects of** ***G. lucidum*** **on Cx43 (A and C) or VEGF (B and D) protein levels in HOCC and HPOC.** *P < 0.05 vs control (0 μg/ml).

**Figure 6:**
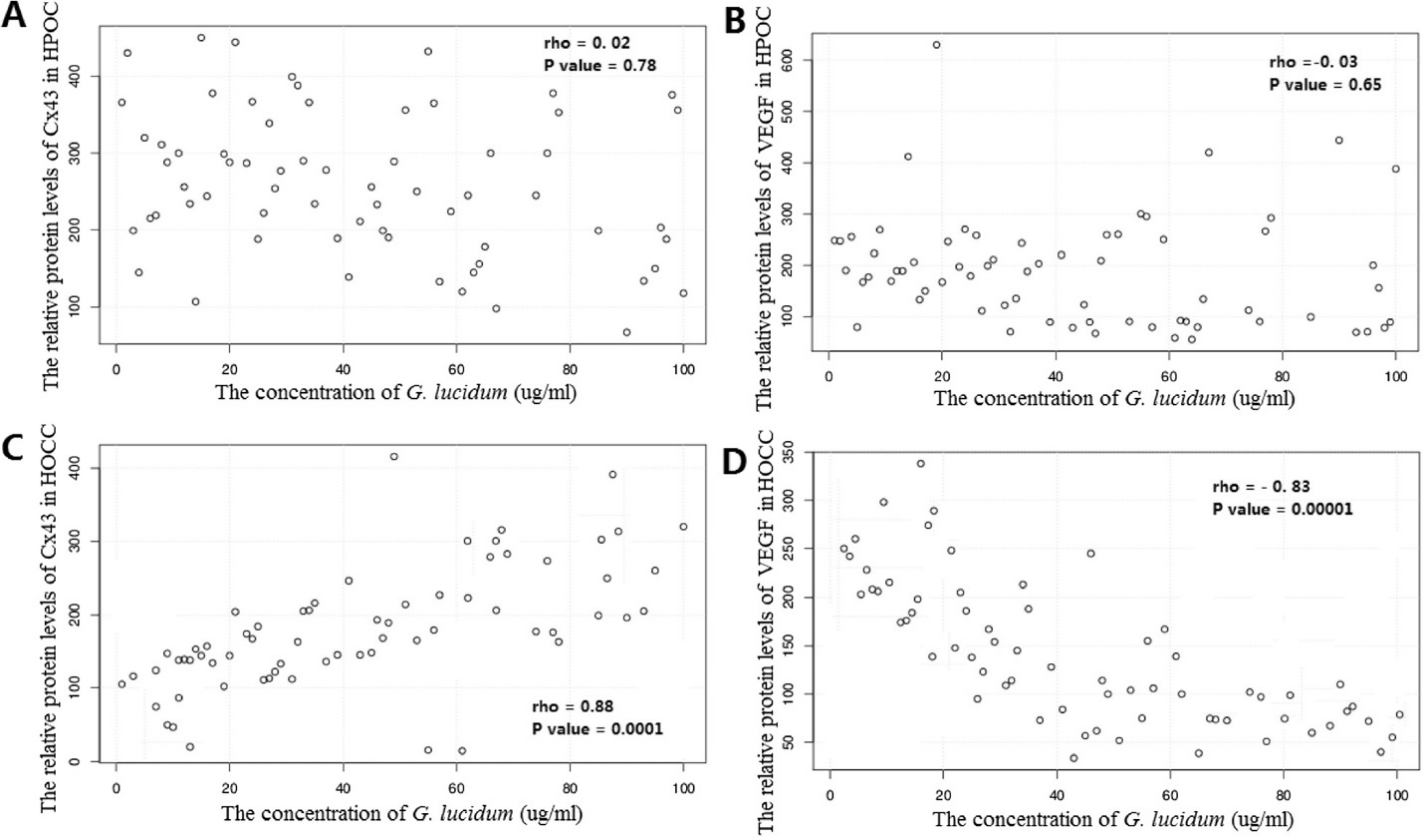
**The relationship between the different concentrations of** ***G. lucidum*** **and Cx43 (A and C) or VEGF (B and D) protein levels in HOCC and HPOC.** Statistical analysis was done by Spearman’s rank correlation test.

In HPOC, increase in *G. lucidum* concentration did not induce significant changes in protein level of Cx43 or VEGF (Figure 5). In contrast, increase in G. lucidum concentration increased protein level of Cx43 and decreased protein level of VEGF in a dose dependent manner in HOCC. Furthermore, Spearman’s rank correlation coefficient showed a significant correlation between the concentration of *G. lucidum* and protein level of Cx43 or VEGF in HOCC but not in HPOC (Figure 6).

### Knockdown of Cx43 abrogates the effect of *G. lucidum*on HOCC proliferation without alteration of *G. lucidum-*induced attenuation of VEGF

**Figure 7:**
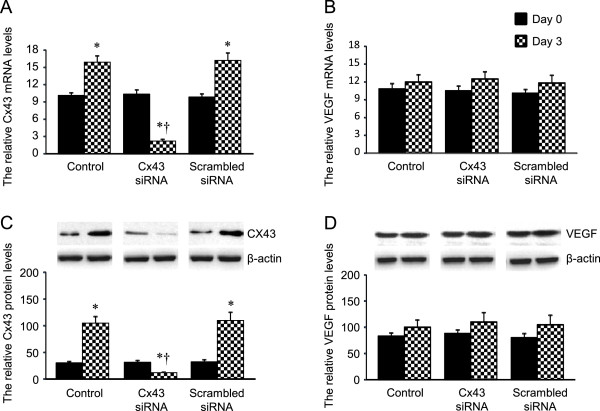
**Effects of Cx43 siRNA or scrambled siRNA on mRNA and protein levels of Cx43 (A and C) and VEGF (B and D) in HOCC treated with** ***G. lucidum.***

**Figure 8:**
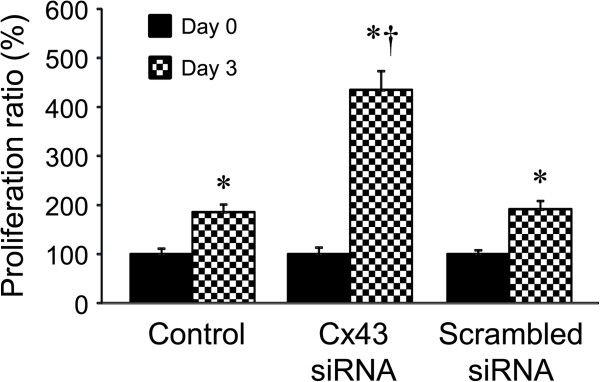
**Effects of Cx43 siRNA or scrambled siRNA on cell proliferation in HOCC treated with** ***G. lucidum.*** Cells were collected before (day 0) and 3 days after co-treatment with *G. lucidum* and siRNAs. Data are shown as mean ± S.D. of three independent experiments. *P < 0.05 vs respective day 0; †P < 0.05 vs corresponding control or scrambled group.

To further explore the molecular mechanism involved in the down-regulation of VEGF expression and up-regulation of Cx43 expression by *G. lucidum*, we examined the effects of knockdown of Cx43 on VEGF expression and cell proliferation in HOCC treated with *G. lucidum*. As shown in Figures 7 and 8, there were no differences in mRNA expression and protein levels for Cx43 and VEGF (Figure 7), and cell proliferation (Figure 8) at day 0. *G. lucidum* treatment significantly increased Cx43 expression, and prevented the expression of VEGF and cell proliferation in control group at day 3. Cx43 siRNA, but not scrambled siRNA, markedly reduced Cx43 expression at day 3 in HOCC treated with G. lucidum. Reduction in Cx43 expression by Cx43 siRNA did not alter *G. lucidum*-induced attenuation of VEGF, but abrogated the beneficial effects of *G. lucidum* on cell proliferation.

## Discussion

Ovarian cancer is the most frequent cause of gynecologic cancer-related death in women [24]. Clinical and pathological diagnosis carried out by various scans and tumor biopsies are key factors for correct and timely treatment [25–27]. Few cancer biomarkers are also currently used for population screening, disease diagnosis, prognosis, monitoring of therapy and prediction of therapeutic response [28].

The anticancer effects of G. lucidum have been attributed to its bioactive compounds including polysaccharides and a group of triterpenes [3]. The precise molecular mechanism by which *G. lucidum* exerts its anticancer properties on ovarian cancer remains incompletely understood. Our present study suggests that *G. lucidum* treatment reduced the expression of VEGF and increased the expression of Cx43, in a dose dependent manner, which was accompanied by decreased cell proliferation in HOCC. More importantly, we found that knockdown of Cx43 with siRNA abrogated the beneficial effect of *G. lucidum* on cell proliferation without altering the *G. lucidum*-induced attenuation of VEGF in HOCC. These findings suggest that VEGF acts downstream Cx43 to mediate cell proliferation. *G. lucidum* exhibits an anticancer effect by up-regulating Cx43 expression via down-regulation of VEGF expression. Previous studies showed that VEGF increased the protein expression of Cx43 in endothelial cells and cardiac myocytes [17, 18]. The discrepancy between our results and others might be due to differences in cell type and treatment method.

Our results indicate that CX43 and VEGF play important roles in the genesis and development of ovarian cancer. Measurements of CX43 and VEGF may also be useful in evaluating effects of treatment. Cx43 is one of the major gap junction proteins which are important for intercellular communication, cell homeostasis and proliferation [29]. VEGF may be a useful serological biomarker for clinical diagnosis and prognosis of ovarian cancer, follow-up of ovarian tumor metastasis and for monitoring the efficacy of therapy in patients with ovarian carcinomas [30]. Thus, the combination of Cx43 and VEGF may provide better diagnosis of ovarian cancer. It is known that healthy subjects have higher expression of Cx43 than patients with ovarian cancer. Our data suggests that Cx43 immunological activity could be used as an ancillary study in the cases that are clinically suspicious for primary ovarian malignancy.

A major limitation of the present study should be acknowledged. The effect of *G. lucidum* on cell proliferation in HOCC was compared with untreated HOCC and HPOC. However, HPOC cells did not proliferate with the same intensity as HOCC cells. Nevertheless, the decreased cell proliferation was clearly observed in *G. lucidum*-treated HOCC, indicating anticancer action of *G. lucidum* in ovarian cancer.

## Conclusions

In conclusion, the present study demonstrates that *G. lucidum* inhibits ovarian cancer by down-regulating the expression of VEGF and up-regulating the downstream Cx43 expression. *G. lucidum* may be a promising therapeutic agent for the treatment of human ovarian cancer.
